# Recent advances in standards for collaborative Digital Anatomic Pathology

**DOI:** 10.1186/1746-1596-6-S1-S17

**Published:** 2011-03-30

**Authors:** Christel Daniel, François Macary, Marcial García Rojo, Jacques Klossa, Arvydas Laurinavičius, Bruce A Beckwith, Vincenzo Della Mea

**Affiliations:** 1 ADICAP - INSERM, UMR_S 872 eq20 Paris, F-75006, France; 2 ASIP Santé, 9 rue Georges Pitard – 75015 Paris, France; 3 Hospital General de Ciudad Real, Department of Pathology, 13005 Ciudad Real, Spain; 4 Tribvn, 39 rue Louveau 92320 Châtillon, France; 5 National Center of Pathology and Vilnius University, P. Baublio 5, Vilnius, Lithuania; 6 North Shore Medical Center, Department of Pathology, 81 Highland Ave. Salem, MA 01970, USA; 7 University of Udine, Department of Mathematics and Computer Science, via delle Scienze 206 33100 Udine, Italy

## Abstract

**Context:**

Collaborative Digital Anatomic Pathology refers to the use of information technology that supports the creation and sharing or exchange of information, including data and images, during the complex workflow performed in an Anatomic Pathology department from specimen reception to report transmission and exploitation. Collaborative Digital Anatomic Pathology can only be fully achieved using medical informatics standards. The goal of the international integrating the Healthcare Enterprise (IHE) initiative is precisely specifying how medical informatics standards should be implemented to meet specific health care needs and making systems integration more efficient and less expensive.

**Objective:**

To define the best use of medical informatics standards in order to share and exchange machine-readable structured reports and their evidences (including whole slide images) within hospitals and across healthcare facilities.

**Methods:**

Specific working groups dedicated to Anatomy Pathology within multiple standards organizations defined standard-based data structures for Anatomic Pathology reports and images as well as informatic transactions in order to integrate Anatomic Pathology information into the electronic healthcare enterprise.

**Results:**

The DICOM supplements 122 and 145 provide flexible object information definitions dedicated respectively to specimen description and Whole Slide Image acquisition, storage and display. The content profile “Anatomic Pathology Structured Report” (APSR) provides standard templates for structured reports in which textual observations may be bound to digital images or regions of interest. Anatomic Pathology observations are encoded using an international controlled vocabulary defined by the IHE Anatomic Pathology domain that is currently being mapped to SNOMED CT concepts.

**Conclusion:**

Recent advances in standards for Collaborative Digital Anatomic Pathology are a unique opportunity to share or exchange Anatomic Pathology structured reports that are interoperable at an international level. The use of machine-readable format of APSR supports the development of decision support as well as secondary use of Anatomic Pathology information for epidemiology or clinical research.

## Introduction

The concept of Collaborative Digital Anatomic Pathology refers to the use of information technology that supports the creation and sharing or exchange of information, including data and images, during the complex workflow performed in an Anatomic Pathology department from specimen reception to report transmission and exploitation. Anatomic Pathology Information Systems (APIS) and digital image acquisition modalities (gross station, microphotography, and virtual microscopy) are two main components of Collaborative Digital Anatomic Pathology but other information systems, like laboratory autostainer’s control software, automated image analysis tools, telepathology systems, biobank management systems are also used in daily practice and contribute to the delivery of diagnostic and prognostic information.

Therefore, achieving Collaborative Digital Anatomic Pathology is a global integrated effort consisting not only in acquiring all the necessary computer equipment and imaging devices needed for the management of the Anatomic Pathology reports and their corresponding images within the hospital, but also in developing architecture that allows collaborative work between different healthcare facilities. Collaborative processes require sharing or exchanging Anatomic Pathology information (data and images) that is unambiguously understandable to human beings. Digitalizing and standardizing this information so that it becomes also unambiguously understandable by machines allows the development of advanced services supporting the interactions between healthcare providers involved in various activities related to patient care coordination as well as epidemiology or clinical research. Collaborative Digital Anatomic Pathology can only be fully achieved using medical informatics standards. The goal of the international integrating the Healthcare Enterprise (IHE) initiative is precisely specifying how data standards should be implemented to meet specific health care needs and making systems integration more efficient and less expensive [1]. The international IHE initiative, developed in North America, Europe and Asia, builds in many healthcare domains, along annual cycles, integration profiles, each of which being an implementable specification of an interoperable solution fulfilling a set of use cases. Each annual cycle is concluded by the organization of international platforms of interoperability tests (called ‘‘connectathons’’) that confer to IHE its unique efficiency. Participation of European researchers in IHE Anatomic Pathology is fostered and partly coordinated by the COST action IC0604 [2]. The results already achieved by IHE Anatomic Pathology, launched in 2005, consist in a technical framework including the integration profile “Anatomic Pathology Workflow” that successfully addresses basic image acquisition and reporting processes within hospitals [3-5].

### Whole Slide Image, emerging technology challenging Collaborative Digital Anatomic Pathology

Anatomic Pathology images – representative “still” images as well as Whole Slide Images – are key information objects of the Collaborative Digital Anatomic Pathology and will become an integral component of Electronic Health Records (EHR) as part of Anatomic Pathology reports [6,7]. “Whole Slide Imaging” is challenging the Anatomic Pathology domain since it offers new promising perspectives for more efficient collaborative practices and also brings some barriers to overcome. Virtual microscopy is already being widely applied in anatomic pathology undergraduate teaching, distance learning and continuing medical education [8], proficiency testing [9,10], quality assurance programs [11-13], research (tumour banking) [14] and teleconsultation (for second opinion). Regarding the latter, the use of Whole Slide Images has been validated for diagnostic applications in surgical pathology [15,16], cytopathology [17], and immunohistochemistry [18,19]. Some automated image analysis algorithms that are being used on digital slides have been U.S. Food and Drug Administration (FDA) approved for assessing the level of certain immunohistochemical markers. However, although there is no statistically significant difference in the diagnostic accuracy either between virtual microscopy and conventional light microscopy, there is little experience in the use of virtual microscopy in diagnostic pathology daily practice, and there is no slide scanner for digital pathology that is FDA cleared for primary or initial diagnosis.

Although Whole Slide Imaging is a promising trend, short term issues have arisen that were challenging standardization organizations with regards to the integration of Whole Slide Images in the Collaborative Digital Anatomic Pathology processes.

### Semantic interoperability of Anatomic Pathology structured reports

Anatomic pathology reports document the pathologic findings in specimens removed from patients for diagnostic or therapeutic reasons. This information can be used for patient care, clinical research and epidemiology. The lack of standards for structuring the relevant data elements in reports, hamper the exchange of this information among different information systems and healthcare organizations. Standardizing and computerizing anatomic pathology reports is necessary to improve the quality of reporting and the exchange of Anatomic Pathology information [20]. Several studies provide recommendations that delineate the required, preferred, and optional elements which should be included in any Anatomic Pathology report, regardless of report types (e.g reporting guidelines in [21,22]). Several national initiatives intend to define standard clinical models for generic Anatomic Pathology Structured Reports (APSRs) (e.g in Germany, the Netherlands or Australasia). Other initiatives focus on specific types of APRs, mainly in the cancer domain. In the United States, the CAP (College of American Pathologists) has published 67 cancer checklists and background information [23]. In France, the SFP (French society of pathology) has published minimum data sets for 21 cancer locations [24]. In Australasia, the Royal College of Pathologists Australasia (RCPA) has published 6 organ specific cancer templates [25]). In some cases implementation guides for these APSR models based on information technology standards (e.g XML) or healthcare information technology standards (e.g HL7 CDA or CEN archetypes) are also provided.

Since these standardization efforts are conducted at a national level, there are some discrepancies between clinical models across countries and even some heterogeneity between clinical models within the same national initiative. Furthermore, although the involvement of Standards Development Organizations such as CEN, HL7 and IHTSDO in joint initiatives addresses some semantic interoperability issues it remains challenging to propose an implementation guide for Anatomic Pathology structured reports providing a formal unambiguous representation of the meaning of Anatomic Pathology observations [26,27].

#### Objective

Our objective was to define the best use of medical informatics standards in the management of Whole Slide Images and Anatomic Pathology structured reports within a Collaborative Digital Anatomic Pathology environment. In the method section we describe the IHE methodology based on working sessions of groups that include both health care providers and information systems vendors who specify technical frameworks in order to support the exchange of information in real-world situations.

In the result section, we first describe how the recently approved DICOM supplements dedicated to Anatomic Pathology allow integrating properly Whole Slide Images to the Healthcare Enterprise. Then we describe the IHE Anatomic Pathology Structured Report (APSR) content profile resulting from a joint IHE and HL7 anatomic pathology activity. This content profile is an implementation guide based on HL7 CDA, a well-established health care standard for electronic clinical document, dedicated to the sharing and exchange of APSRs across healthcare facilities.

Lastly, we discuss the benefits and challenges of standardizing the use of both Whole Slide Images and structured reports in a Collaborative Digital Anatomic Pathology environment. We especially discuss the ongoing process of specifying the use of SNOMED CT concepts in order to formally represent Anatomic Pathology observation in structured reports.

## Material, methods

We used the methodology of the Integrating the Healthcare Enterprise (IHE) initiative which has been developed in North America, Europe, and Asia. The IHE process is based on working groups that include both health care providers who define precise users’ needs and information systems vendors in charge of defining domain-specific “integration profiles”, i.e. standard-based exchange of information in real-world situations. Integration profiles describe informatics transactions leveraging and constraining established industry standards such as DICOM or HL7. The annual definition cycle of new profiles by users and suppliers - ending in the organization of international platforms of interoperability tests (called ‘‘connectathons’’) - confers its unique efficiency, transforming basic standards into ‘‘plug and play’’ solutions.

### Working Groups and Sessions

The sponsors of the IHE initiative in the Anatomic Pathology domain (ADICAP- Association pour le Développement de l’Informatique en Cytologie et Anatomie Pathologiques, France, SEAP - Spanish Society of Pathology, Spain, SEIS - Spanish Society of Health Informatics, Spain, CAP - College of Amercian Pathologists, USA.) solicited practicing pathologists and hematologists; information technology professionals; and vendors from France, Spain, Italy, Germany, Japan and the United States to work on the IHE Anatomic Pathology technical framework. The IHE Anatomic Pathology working group conducted 23 working sessions between September 2005 and June 2010 (approximately one meeting every three months). Some of these meetings were supported by the COST action IC0604, funded by the European commission, within the working groups WG1 (business modeling) and WG2 (IT standard) [28].

If errors in existing standards or the need for extensions are identified, IHE’s policy is to report them to the appropriate standards bodies (HL7 or DICOM) for resolution within their conformance and standards evolution strategy. American, European, and Japanese groups agreed that, although specific DICOM objects were defined for Anatomic Pathology digital images, modification and/or extension were necessary for two main reasons. First, the DICOM model did not initially describe specimens in sufficient detail or associate images with specimens with enough precision for the complexity of Anatomic Pathology practice; and second, some pathology-related image formats (Whole Slide Images, multispectral images, flow cytometry, etc) did not have applicable DICOM information object definitions. To address these issues, a specific DICOM pathology working group (WG26) was created in December 2005 and several IHE Anatomic Pathology–DICOM WG26 joint working sessions have been organized [29].

Meanwhile, the HL7 Anatomic Pathology WG was established to investigate the complex relationships between specimens, observations, images and documents in Anatomic Pathology. Joint meetings between the IHE Anatomic Pathology and HL7 Anatomic Pathology working groups have also been regularly conducted [30].

### Integrating Whole Slide Images to the healthcare enterprise

Since Anatomic Pathology is a specimen-centric process, a first activity conducted across the IHE, HL7 and DICOM working groups dedicated to Anatomic Pathology was to agree on a specimen model, i.e. the way to identify and describe specimens that are the subject of one or more procedure steps in the workflow including imaging procedures. Another key issue was to provide a DICOM information object definition applicable to Whole Slide Images.

### Integrating Anatomic Pathology structured reports to the healthcare enterprise

As part of joint IHE and HL7 anatomic pathology activities, an ongoing effort consists in defining a formal information model for Anatomic Pathology Structured Report (APSR) based on HL7 Clinical Document Architecture (CDA) allowing binding textual information to images or regions of interest [31]. Based on the review of published recommendations and national or international initiatives providing standard clinical models for APSRs, the first step for international experts was to agree on the data structure of a generic clinical model for APSRs and for the set of constraints that apply specifically to cancer APSRs (whatever the organ is) and even more specifically to some organ-specific cancer APSRs. The second step was to agree on the format to be used to computerize these APSRs and to provide the implementation guide based on this format. The last step was to define the use of coding systems to encode anatomic pathology observations in APSR templates. The most frequently used coding systems in anatomic pathology domain are SNOMED Clinical Terms®, ICD-O-3 and ADICAP in France.

## Results

IHE Anatomic Pathology activities resulted in the definition of IHE integration and content profiles supporting and standardizing Collaborative Digital Anatomic Pathology processes and especially defining the use of “Whole Slide Images” and semantically interoperable structured reports within these processes.

### Flexible information object definitions dedicated to whole slide image acquisition, storage and display

During the fulfillment of an order of Anatomic Pathology examination, some imaging procedure step(s) may be performed by acquisition modality(ies) on specimen (gross imaging) or derived specimen (smears or tissue sections)(microscopic imaging, including Whole Slide Imaging). DICOM defines a hierarchy of concepts related to medical imaging workflow in order to precisely organize the digital images related to the same order. The highest level is the *study*, which, for Anatomic Pathology, contains all information (images and text) collected in the process of fulfilling a given order. The study comprises one or more *series*. Each series contains one or more images. Each study may contain images acquired by different modalities (gross imaging, microscopic imaging, etc). Whenever an image is acquired from a new specimen or involves a new acquisition modality a new series is created. A new series may also be created when an image is acquired for an existing study after the original order has been fulfilled.

Two **DICOM supplements** were defined by the DICOM WG26 in order to better address the specificity of information objects in the Anatomic Pathology domain.

#### DICOM supplement 122

**Figure 1:**
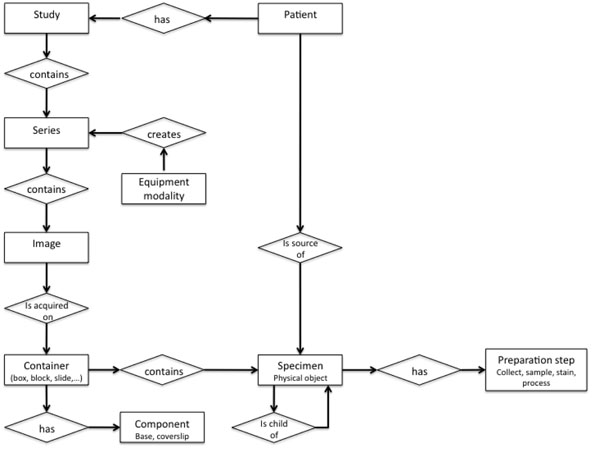
DICOM Model of the Real World” extended for specimen with the addition of the objects “specimen,” “container,” “component,” and “preparation step.”

Existing information object definitions (IODs) previously defined by DICOM for Anatomic Pathology - *visible light photographic image* for gross specimens and *visible light slide-coordinates microscopic image* for slide-based microscopic imaging - did not have a strong mechanism for describing the specimen being imaged or associating a particular specimen with a particular image. In fact, while the relationship between patient and image is straightforward in other imaging fields and accurately captured by DICOM objects, in Anatomic Pathology there was a need for a new robust specimen module to formally define specimen attributes at the image level. The **DICOM supplement 122** defines formal DICOM attributes for the identification and description of specimens when said specimens are the subject of a DICOM image [32]. In this supplement, the “DICOM Model of the Real World” has been extended for specimen with the addition of the objects “*specimen*,” “*container*,” “*component*,” and “*preparation step*” (figure 1). Attributes of the specimen, container, component, and preparation step objects are represented in the specimen module, which is focused on critical specimen information necessary to interpret the image. Specimen attributes include attributes that (1) identify the specimen (within a given institution and across institutions); (2) identify and describe the container in which the specimen resides as well as each component of the container if required (e.g. a “slide” is a container that is made up of the glass slide, the coverslip, and the “glue” that binds them together); (3) describe specimen collection, sampling, and processing; and (4) describe the specimen or its ancestors when these descriptions help with the interpretation of the image. The specimen module distinguishes the container ID and the specimen ID, making them different data elements to allow maximal flexibility for different situations. Even though the full history of specimen processing is not required in every instance, specimen attributes allow that processing history to be encoded. Attributes that convey diagnostic opinions or interpretations are not within the scope of the specimen module. The DICOM specimen module does not seek to replace or mirror the pathologist’s report. The DICOM specimen module has been harmonized with the HL7 v2 SPM segment and the HL7 v3 specimen domain information model. Some hospitals already started to work with digital slides identified by means of Supplement 122 techniques [33,34].

#### DICOM supplement 145

**Figure 2:**
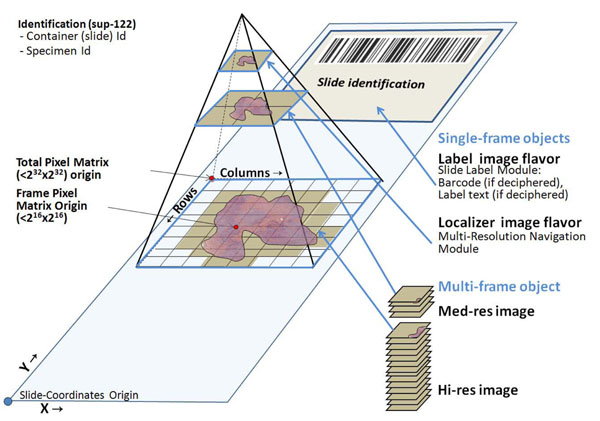
Whole Slide Image Information Object Definition (WSI IOD) from DICOM supplement 145 proposes storing tiles from a multi resolution hierarchy in multi-frame object(s). Each tile is stored in a Frame and is located within a 2^32^x2^32^ total pixel matrix. Specific Z planes or/and optical paths may be specified at the frame level.

Another key issue was to define DICOM information object definition (IOD) applicable to Whole Slide Images. Whole Slide Images are different from traditional microphotographs in multiple ways. First, they are considerably larger, and this large size prevents the usual paradigm of “store and forward” to be used for Whole Slide Images. Second, for performance reasons, Whole Slide Images are usually accessed remotely using an image browser which only loads a small portion of the overall image pixel data. In addition, the need for displaying these images at multiple different “magnifications” is another technical and architectural challenge. The proposed **DICOM supplement 145**[35] deals with all of these issues and tries to provide the maximum amount of flexibility to image acquisition, storage and display devices and software. For a variety of reasons, the proposal introduces the concept of tiling (breaking down the full image into multiple smaller images which can be handled separately) for storage of Whole Slide Images. However, images which are smaller than the current image size limits in DICOM can also be stored as JPEG2000 images and accessed via the JPIP protocol, both of which are supported by DICOM already. In addition, the proposed IOD has provisions for handling multi-spectral images, multiple focal planes and other necessary features, as well as allowing for detailed descriptions of the optical components used to create the image (Figure 2). A system compliant with Supplement 145 will be able to store digital slides directly on a PACS, while a compliant viewer will be able to retrieve slides directly from a PACS.

### Semantically interoperable Anatomic Pathology structured reports

The IHE “Anatomic Pathology Structured Report” (APSR) content profile is an implementation guide based on HL7 CDA dedicated to the sharing and exchange of APSRs across healthcare facilities. HL7 CDA provides a general architecture for designing and implementing clinical documents in an electronic format that is both human and machine-readable. Because of the architectural nature of the CDA standard, individual implementations are always associated with an implementation guide (also called “HL7 CDA template”), i.e. a document that describes how the CDA standard should be implemented for a particular type of document used in a specific context.

The current scope of the IHE “Anatomic Pathology Structured Report” (APSR) content profile is surgical pathology. It addresses all fields of Anatomic Pathology (cancers, benign neoplasms as well as non-neoplastic conditions) but handles information of only “traditional” Anatomic Pathology observation using light microscopy (including immunohistochemistry, FISH, etc)). Cytopathology, forensic medicine (autopsy, toxicology) will be addressed in further cycles as well as special ancillary techniques (flow cytometry, cytogenetics, and electronic microscopy).

A CDA document begins with a header that states the context of care in which the document was produced, identifies the various participants involved (patient, care providers, devices, etc) and states the responsibilities regarding the content of the document. The body of the document can be organized as a hierarchy of sections. Each section lays out its text for the reader, and may in addition carry fine-grained coded machine-readable data, corresponding to that text.

**Figure 3:**
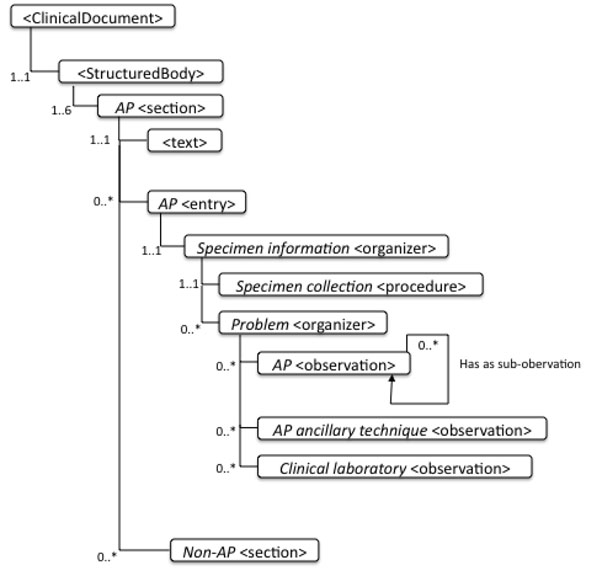
Common structure of all APSR documents. In each section, observations are organized per specimen and per problem.

A generic HL7 CDA template has been designed to address structured reporting whatever the organ and diagnosis are. Specialized templates address more specific items dedicated for example to cancer structured reports. Templates were specified for 20 organ-specific cancer APSRs. HL7 CDA templates include required data elements, as well as optional ones, that can be further specified as required in national extensions. We first defined 6 body sections (Clinical Information Section, Intraoperative Observation Section, Macroscopic Observation Section, Microscopic Observation Section, Diagnosis Section, Procedure step Section), and assigned each section a unique code, a title and a text block. We coded the fine-grained machine-readable data into entries attached to the sections. We defined entry elements and especially 68 Anatomic Pathology observations and 12 Anatomic Pathology ancillary techniques observations. Figure 3 describes how observations are organized in sections per specimen and per problem (thanks respectively to the specimen information organizer and the problem organizer).

#### Binding Anatomic Pathology observations to their evidences

Since it is useful to present to the reader of the report the images related to the observations, HL7 CDA templates of APSRs provide means to embed images at the observation level or at the organizer level in an entry, using the *observationMedia* element and potentially the *regionOfInterest* element. These images can only be small illustrations. Big images – like Whole Slide Images or evidence documents – will stay in their own storage infrastructure, and may be associated with the APSR document using reference to an external observation (via a DICOM KOS list of references).

#### Using coding systems to encode anatomic pathology observations

Entry elements in APSR templates are of different data types: Integer, Time Stamp, Encoded Data, (which supports multimedia), Interval of Time, Coded with Equivalents, Concept Descriptor, etc. The two last data types can carry Concept identifiers. Coded with Equivalents (CE), carries a code, the name of the coding scheme the code is drawn from, and a display name corresponding to the code; and allows synonyms to be transmitted – such as a SNOMED CT code and its equivalent ICD-O code or ADICAP code. Concept Descriptor (CD) adds the support for post-coordination of codes (i.e the combining of codes from a terminology to create a new concept). At the level of the Reference Information Model (RIM), attributes of type CE or CD will declare a single “vocabulary domain”. Some of these vocabulary domains are internally defined by HL7 V3. The CDA standard and CDA template specifications further restrict this vocabulary domain. A vocabulary domain can be constrained to a well defined set of acceptable codes taken from one or more coding systems (such as LOINC, SNOMED CT, ICD-10) creating a “value set” bound to the CE or CD attribute.

We coded the fine-grained machine-readable data into entries attached to the sections. Codes have been assigned to sections and to the various entry elements (acts (observations, procedures, etc), entities (specimen)) carried within the entries. For some of the CDA elements, the vocabulary domain is imposed by the standard. For others, the implementer is free to choose from any relevant external source, such as LOINC, SNOMED CT or some other realm-specific vocabulary. LOINC codes were found to encode the document type and the sections. For anatomic pathology observations and AP ancillary technique observations, we defined a coding system dedicated to the IHE Anatomic Pathology domain (PathLex - OID : 1.3.6.1.4.1.19376.1.8.2.1).

We provided PathLex codes for 68 Anatomic Pathology observations and 12 Anatomic Pathology ancillary techniques. For the 43 observations of type CD or set<CD>, we defined 266 value sets and provided codes for the 1488 values of these value sets. The terms and expressions of PathLex are being currently mapped to SNOMED CT concepts. In national extension, the vocabulary domain may be specifically constrained. For example, the possible values for the observation “histological type”, encoded using PathLex value sets, will be also encoded in France using ADICAP value sets.

#### Sharing or exchanging APSRs across healthcare facilities

The Anatomic Pathology report, as an HL7 CDA conformant document, may be published towards a document sharing resource such as an Electronic Health Record (EHR) or Personal Health Record (PHR). The Cross-Enterprise Document Sharing (XDS) integration profile defined in the IHE Information Technology Infrastructure (ITI) Technical Framework, enables a number of healthcare delivery organizations belonging to an XDS Affinity Domain (e.g. a community of care) to cooperate in the care of a patient by sharing clinical records in the form of documents as they proceed with their patients’ care delivery activities. Federated document repositories and a document registry create a longitudinal record of information about a patient within a given XDS Affinity Domain. This profile is based upon the ebXML Registry standards from OASIS, and a number of standards from W3C (SOAP, HTTP, etc). It describes the configuration of an ebXML Registry in sufficient detail to support Cross Enterprise Document Sharing.

In addition, physical media may be used to carry the Anatomic Pathology report or this report may be conveyed using person-to-person email. Cross-Enterprise Document Media Interchange (XDM) integration profile provides document interchange using a common file and directory structure over several standard media.

With regards to image integration in the reporting solutions, as already stated, big images – like Whole Slide Images – will stay in their own storage infrastructure, and may be associated with the APSR document using reference to an external observation (via a DICOM KOS list of references (as described in the XDS-I profile from the Radiology domain)). Therefore the Content Consumer application must support the DICOM protocol to access the images.

## Discussion and conclusion

The main output of Collaborative Digital Anatomic Pathology is a timely and clear report of diagnostic and prognostic information crucial to patient care, clinical research and epidemiology, which is more and more developed in a collaborative way, involving various professionals, various techniques, and various evidences. Digital images and especially Whole Slide Images offer new promising perspectives for Collaborative Digital Anatomic Pathology, being themselves evidence of what is described in the report and/or the basis for producing further evidence by other pathologists or by image analysis software.

This paper describes first how Whole Slide Image management can be closely integrated to the information flow of Collaborative Digital Anatomic Pathology using existing and emerging medical informatics standards like DICOM (especially DICOM supplements 122 and 145) and HL7 following the recommendations of Integrating the Healthcare Enterprise (IHE) Anatomic Pathology technical framework. A key result is the DICOM supplement 145, which enables a compliant system to store Whole Slide Images directly on a PACS, and a compliant viewer to retrieve them from there, which should greatly simplify the use of such images in a variety of settings. Indeed, the main advantage of using the DICOM standard instead of proprietary file formats is to store anatomic pathology images in PACS, like radiologists or cardiologists do, and therefore to distribute these images to clinicians through the same viewers that they use for other medical images.

A main contribution of the joint IHE and HL7 Anatomic Pathology collaboration is the on-going effort to define international content profile for surgical Anatomic Pathology Structured Reports (APSR) including specialized models for generic cancer APSR and organ/procedure specific cancer. This content profile is a unique opportunity to provide world-wide unified solutions for anatomic pathology reporting and especially cancer reporting. The main issue is that although HL7 CDA international implementation guides express a minimal set of internationally required and standardized observations they should remain flexible enough to take into consideration national or local constraints (e.g. local coding systems, local value sets) within national extensions. With regards to image integration in the reporting solutions, the technical options offered by CDA templates to address this issue for both small illustrative images and big information objects – like Whole Slide Images or evidence documents – require new advanced developments from both acquisition modalities and PACS (e.g implementation of the IHE XDS-I profile).

Another challenging issue is achieving semantic interoperability of APSRs. Although it is, in general, straightforward to use SNOMED CT concepts to encode observations (e.g histologic type), for some other observations (e.g TNM codes) it remains challenging to use SNOMED CT in order to constrain vocabulary domains attached to these observations.

Although Standards Development Organizations such as CEN, HL7 and IHTSDO [36] in joint initiatives addresses some semantic interoperability issues and provides a better understanding of the gaps and overlaps in semantics at the interface of HL7 and SNOMED CT, clear guidelines supporting SNOMED CT encoding of HL7 attributes are not yet available.

An important complication is that information systems operate at two different levels, which Rector et al. describe as “models of use” and “models of meaning” [37,38]. The model of use describes how information system data is actually represented (e.g in Anatomic Pathology Information Systems, Electronic Healthcare Records or Clinical Data Warehouses), including the way that data are captured and displayed (e.g through APSR templates). The “model of meaning” represents our understanding of the world so that both human and computers can reason about it. It provides information in a common, standardized format for data processing and reasoning. On going efforts are conducted to bind EHR reference models, such as HL7 Reference Information Model (RIM), the EN13606-1 or the openEHR reference model, to reference terminologies such as SNOMED CT.

As part of the “model of use”, the so-called “interface terminologies” containing relatively common clinical terms are designed to improve acceptability of information systems to healthcare providers. In the area of patient care, Rosenbloom et al. condensed various interface terminology definitions to produce the following: “systematic collections of clinically oriented phrases (i.e., ‘terms’) aggregated to support clinicians’ entry of patient information directly into computer programs, such as clinical documentation (i.e., ‘note capture’) systems” [39,40].

Despite their prevalence for electronic data capture, no single standard interface terminology exists. In contrast, standards have been identified for reference terminologies such as SNOMED CT, the emerging global health terminology standard published by IHTSDO, that provides unified meanings for clinical terms from different languages by assigning them to language-independent concepts. Furthermore, reference terminologies are typically optimized to support the storage, retrieval, and classification of clinical data. Mapping interface terminologies (as part of a model of use) to standard reference terminologies (as part of the model of meaning) rather than identifying one or more interface terminologies to serve as standards is now a commonly admitted strategy towards semantic interoperability [41].

The coding system dedicated to the IHE Anatomic Pathology domain (PathLex) acts as an “interface terminology” is currently being mapped to SNOMED CT concepts since an important pre-requisite to the best implementation of Collaborative Digital Anatomic Pathology is to provide the model of meaning corresponding to the data & images that are captured, shared and exchanged. Using a reference terminology such as SNOMED CT offers promising perspectives in terms of scalable semantic queries that could be performed over distributed Anatomic Pathology Information Systems (APIS), EHRs or Clinical Data Warehouses storing these structured reports.

## Competing interests

The authors declare that they have no competing interests.
